# Terpenoids in the Essential Oil and Concentrated Aromatic Products Obtained from *Nicotiana glutinosa* L. Leaves

**DOI:** 10.3390/molecules25010030

**Published:** 2019-12-20

**Authors:** Venelina Popova, Tanya Ivanova, Albena Stoyanova, Violeta Nikolova, Tsveta Hristeva, Velizar Gochev, Yonko Yonchev, Nikolay Nikolov, Valtcho D. Zheljazkov

**Affiliations:** 1 Department of Tobacco, Sugar, Vegetable and Essential Oils, University of Food Technologies, 4002 Plovdiv, Bulgaria; 2 Tobacco and Tobacco Products Institute—Bulgarian Agricultural Academy, 4108 Markovo, Bulgaria; 3 Department of Biochemistry and Microbiology, Plovdiv University “Paisii Hilendarski”, 4000 Plovdiv, Bulgaria; 4 Department of Crop and Soil Science, Oregon State University, Corvallis, OR 97331, USA

**Keywords:** *Nicotiana glutinosa* L., essential oil, extracts, diterpenes, sclareol, antimicrobial activity

## Abstract

*N. glutinosa* L. is a relatively less studied *Nicotiana* species (Solanaceae), although there are data about its importance as a model plant in viral control studies, as a gene donor in tobacco hybridization and as a source of agents with insecticidal or fungicidal effects. The biological activities of the species were associated mostly with the presence of leaf surface metabolites, in particular diterpenes and sucrose esters. The aim of this study was to identify the chemical composition of the essential oil (EO) and two aromatic extraction products (concrete and resinoid) obtained from *N. glutinosa* L. leaves. GC-MS analysis identified 26 components in the EO (representing 97.3% of total oil content), which contained mostly diterpene compounds with major components manool (14.2%), sclarene (8.4%) and manoyl oxide (8.1%). The number of compounds identified in the concrete was 37 (95.5% of the total content) and the major component was the diterpene alcohol sclareol (14.2%). In the resinoid, 30 volatile components (representing 95.1% of resinoid content) were identified, with major components nicotine (32.9%), α-tocopherol (8.2%), tridecanoin (6.9%), sclareol (6.9%), and solanone (6.9%). The group of bicyclic diterpenes had the largest share in the diterpene fraction of the products (57.3%, 91.7%, and 86.3%, respectively for the EO, concrete, and resinoid). Considering the abundance of sclareol in the aromatic products, the antimicrobial activity of the pure substance was determined. Sclareol was highly effective against a set of medicinally important yeasts; *Candida albicans* АТСС 10231, *C. glabrata* ATCC 90030, *C. parapsilosis* clinical isolate, and *C. tropicalis* NBIMCC 23, while being less effective against the studied Gram-positive and Gram-negative bacteria. Data from the study on *N. glutinosa* aromatic products composition may be of interest to the aroma industries for their possible use in perfumery and cosmetics.

## 1. Introduction

*Nicotiana glutinosa* L. is one of the four *Nicotiana* species (Solanaceae) described by Linnaeus (1753), together with *N. tabacum* L., *N. rustica* L. and *N. paniculata* L. In some early references [1] *N. glutinosa* was regarded as a member of Nicotiana section Tomentosae, although an apparent mixture of traits characteristic of other sections was also noticed. In the early 20th century, researchers already treated *N. glutinosa* as a member of Nicotiana section Undulatae [2,3,4,5].

*N. glutinosa* is native to Northern and Central Peru and Southern Ecuador, where it has adapted well to semi-arid areas, rocky slopes and ditch banks [1,5]. Plants are more fragile and with tender stem than common tobacco (*N. tabacum*), rarely branching [6]. Leaves are petiolate, heart-shaped; with a maximum length of about 23 cm. Inflorescences are racemose with long peduncles. Unlike *N. tabacum*, the flowers of *N. glutinosa* tend to form bilabiate corolla and are deep orange in color with long anthers adhering to the upper lobe of the corolla, as in the bilabiate flowers of other families [6]. *N. glutinosa* germinates readily and grows well in both a greenhouse and the field [7]. There are three accessions of *N. glutinosa*, labeled as accessions (acc.) 24, 24A, and 24B [8].

Data on the chemical composition of *N. glutinosa* leaves are generally limited [5], with the exception of those for alkaloids, as well as for sucrose esters, diterpene alcohols and other leaf surface exudate components [8,9,10,11,12,13,14,15]. The major alkaloid in *N. glutinosa* is nornicotine [16], although some inconsistent alkaloid transformations were observed in the species [12]. The study of the alkaloid composition of 64 *Nicotiana* species [12] found that the total alkaloid content of *N. glutinosa* freeze-dried leaves from greenhouse-grown plants was 7.4 mg/g (of which 77.4% nornicotine and 18.9% nicotine), while the green leaves of field-grown plants, with a similar 7.35 mg/g total alkaloid content, had nicotine as the dominant alkaloid (85.4%), followed by nornicotine (11.3%). The alkaloid distribution in air-cured leaves from filed-grown plants, however, was in a reversed order, 88.1% nornicotine and only 1% nicotine. Sugar esters (mainly sucrose and to a lesser extent glucose esters of different structural types) have been identified as leaf surface components from *Nicotiana* species, including *N. glutinosa*, and their inhibitory effect against different pathogens and insects has been reported [5,8,13,15,17,18,19]. *N. glutinosa* was not associated with the production of cuticular duvane diterpenes, which are characteristic for *N. tabacum,* but it was found to metabolize various labdane diterpenes [20]. Indeed, *N. glutinosa* is denoted as an important natural source of sclareol, together with three other species belonging to different plant families; *Salvia sclarea* L. (Lamiaceae) [21], *Cistus creticus* L. (Cistaceae) [22], and *Cleome spinosa* L. (Brassicaceae) [23]. Labdenediol and sclareol were the major diterpenes in the leaves of *N. glutinosa* accession 24A [14], while another study [24] found the following pattern in the production of cuticular labdanes by the three *N. glutinosa* accessions: acc. 24 produced manool, 2-oxymanool, 2-hydroxymanool, sclareol, 3-episclareol, and labdenediol; acc. 24A produced only sclareol and labdenediol, and the third accession, 24B, did not produce labdanes. In another study [8] the levels of major cuticular components identified in *N. glutinosa* accessions were: acc. 24—labdenediol (6 μg/cm^2^), manool (7 μg/cm^2^), hydroxymanool (23 μg/cm^2^), oxymanool (5 μg/cm^2^), and sclareol (43 μg/cm^2^); acc. 24A—labdenediol (50 μg/cm^2^) and sclareol (94 μg/cm^2^). All parts of *N. glutinosa* plants were capable of accumulating large quantities of sclareol [11]. Still, higher concentrations were found in the upper, younger plant tissues (upper leaves and flower buds), exceeding 600 μg/g tissue.

The economic importance of *N. glutinosa* is connected mainly to its use in tobacco hybridization and as a model plant in viral control studies [25]. *N. glutinosa* showed good potential as a source of biorational agents against whitefly (Hemiptera: Aleyrodidae) [17,26]; anthracnose, *Colletotrichum lagenarium* [27]; blue mold, *Peronospora tabacina* [9]; rust diseases, *Alternaria brassicocola* [10]; powdery mildew of tobacco, *Erysiphe cichoracearum* DC. [28]; black shank, *Phytophtora parasitica* var. *nicotianae* [29], and others [30]. The antifungal and insecticidal effects were strongly related to the concentration of leaf exudate metabolites, and in particular to diterpenes (sclareol, labdenediol, cis-abienol, 13-epi-sclareol, etc.) and sucrose esters.

To the best of our knowledge, there are no previous reports about obtaining natural aromatic products from *N. glutinosa* leaves, i.e., essential oil (EO), concrete, and resinoid, as well about their volatile composition. The last decades, however, have been witnessing an intensive search for novel plant sources to expand the range of natural EOs and extraction concentrates from aromatic and medicinal plants available to meet the ever-increasing demands of the fragrance market. Common tobacco (*N. tabacum*) and some other *Nicotiana* species (for example, its Australian relative *N. benthamiana* Domin) are legitimate medicinal plants, and tobacco absolute, concrete, and supercritical fluid extracts are widely used in fine perfumery and cosmetics [31,32,33]. Therefore, it seemed rational to attempt a similar look on *N. glutinosa*. We hypothesized that the identification of the chemical composition of *N. glutinosa* aromatic products would reveal a specific profile, different from that of other *Nicotiana* species, which would be of potential interest for the fragrance and other industries. Since metabolite accumulation in a given plant species is strongly influenced by environmental factors, the new data from this study on the aroma-related composition of *N. glutinosa* experimentally produced under the ecological conditions of Bulgaria could be a significant contribution to the complex characterization of the species. We further hypothesized that the determination of the antimicrobial activity of the labdane diterpene alcohol sclareol, as a characteristic metabolite of *N. glutinosa*, against selected medicinally important yeast and bacterial strains would expand the existing knowledge about its biological effects and use potential.

Therefore, the aim of this study was to identify the volatile profile, with a special emphasis on diterpenes, of three natural aromatic products (EO, concrete, and resinoid), obtained from *N. glutinosa* leaves produced in Bulgaria, as well as the antimicrobial activity of sclareol against some medically important yeasts and bacteria.

## 2. Results

**Figure 1:**
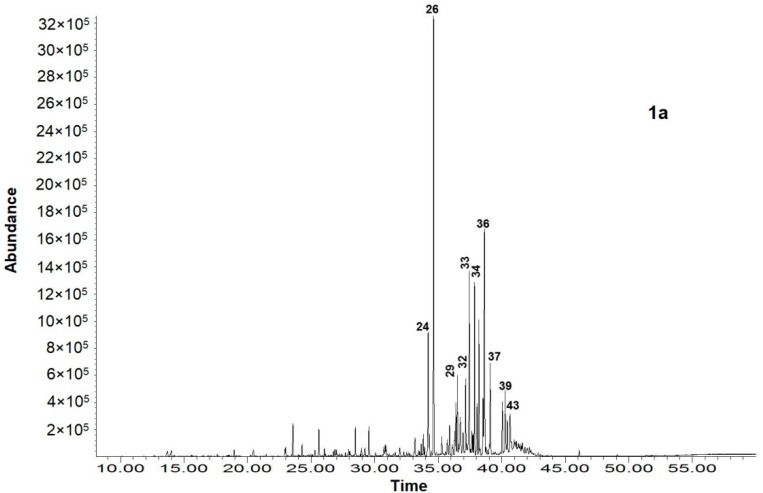

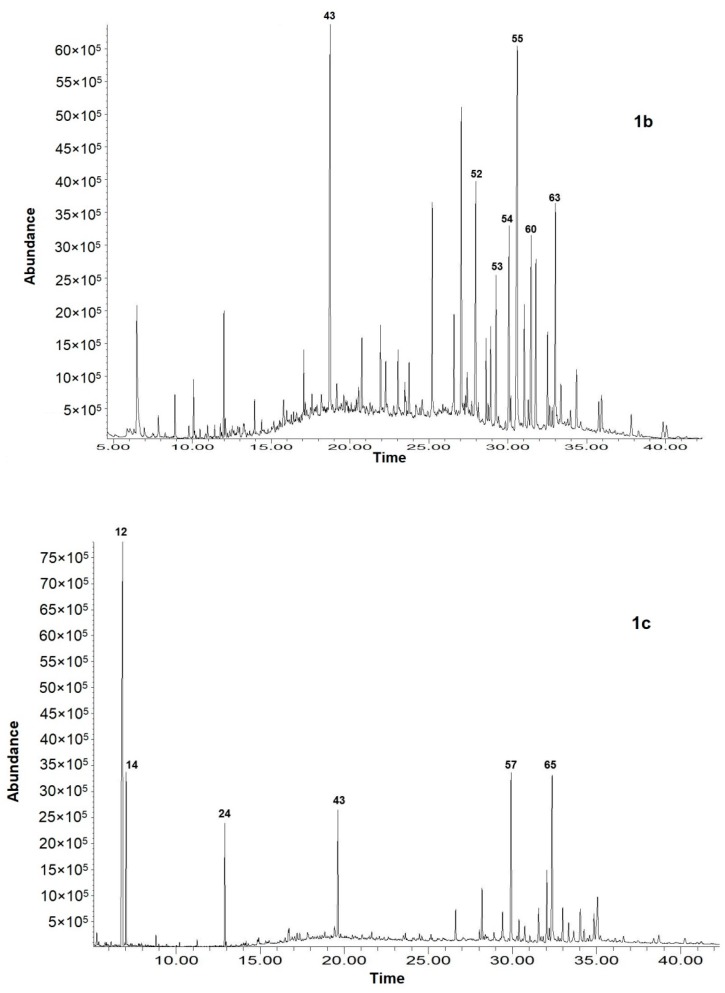
Total ion current (TIC) chromatograms of *N. glutinosa* essential oil (**1a**), concrete (**1b**) and resinoid (**1c**); the major compounds labeling is according to their numbers in Table 1, as follows: 12—nicotine; 14—solanone; 24—hexahydrofarnesyl acetone; 26—diisobutyl phthalate; 29—isopimara-9(11),15-diene; 32—cembrene; 33—sclarene; 34—manoyl oxide; 36—manool; 37—verticillol; 39—pimara-7,15-dien-3-one; 43—sclareol; 52—*n*-octacosane; 53—squalene; 54—*n*-nonacosane; 55—*n*-triacontane; 57—α-tocopherol; 60—*n*-tritriacontane; 63—*n*-hexatriacontane; 65—tridecanoin.

The moisture content of the initial plant sample was 9.2 ± 0.05%. The total ion current (TIC) chromatograms from the GC-MS analysis of the EO, concrete and resinoid isolated from *N. glutinosa* leaves are presented on Figure 1, and the results from the identification of the respective volatile and semi-volatile components, listed in the order of their retention indices, are presented in Table 1. The relative distribution of the compounds identified in the three aroma products, by major groups of CG-MS volatiles, is shown in Table 2 (the total sum of the identified compounds = 100%).

The yield of the EO was 0.23 ± 0.50% DW. The EO was a light yellow liquid, and the olfactory evaluation described it as having a typical green odor. The GC-MS analysis identified 26 components of the EO of *N. glutinosa* leaves, constituting 97.3% of the total content. Eighteen of them were in concentrations over 1% and the rest eight constituents were in concentrations under 1%. The major constituents of the EO (over 3% of TIC) were as follows: diisobutyl phthalate (17.6%), manool (14.2%), sclarene (8.4%), manoyl oxide (8.1%), hexahydrofarnesyl acetone 6.0%), cembrene (5.6%), verticillol (4.8%), 13β-methyl-13-vinyl-podocarp-7-en-3β-ol (4.7%), 13β-methyl-13-vinyl-podocarp-7-en-3-one (4.4%), sclareol (3.6%), dibutyl phthalate (3.5%), and pimara-7,15-dien-3-one (3.4%).

The yield of *N. glutinosa* concrete was 1.56 ± 0.13% DW. In appearance, it represented a light brown viscous mass. The integrated olfactory description of the concrete was “a slightly tobacco odor, with green and earth undertones”. The identification of volatile compounds revealed 37 components responsible for 95.5% of the total content. Twenty-two of them were in concentrations over 1% and the remaining 15 constituents were in concentrations under 1%. The major constituents (more than 3% concentration) of the concrete were as follows: sclareol (14.2%), *n*-triacontane (10.7%), *n*-nonacosane (6.96%), *n*-hexatriacontane (6.5%), *n*-tritriacontane (6.2%), *n*-dotriacontane (5.9%), *n*-octacosane (5.1%), *n*-tetratriacontane (4.1%), trimethylsilyl octanoate (3.9%), *n*-hentriacontane (3.9%), sclareol oxide (3.7%), and *n*-pentatriacontane (3.2%).

The yield of resinoid from *N. glutinosa* leaves, respectively, was 10.6 ± 0.09% DW. The resinoid was a dark brown viscous mass, and the olfactory evaluation resulted in the description of a typical tobacco odor, with green, honey-like, and balsamic undernotes. Thirty components in total were identified in the resinoid, equal to 95.1% of its content. Fifteen of them were in concentrations over 1% and the rest 15 constituents were in concentrations under 1%. The major constituents (over 3%) of the resinoid were as follows: nicotine (32.9%), α-tocopherol (8.2%), tridecanoin (6.9%), solanone (6.9%), sclareol (6.9%), hexahydrofarnesyl acetone (6.5%), and *n*-hentriacontane (3.1%).

The profile of the EO, by groups of chemical compounds, was shaped mostly by diterpenes (61.5%), followed by phenyl propanoids (24.6%) and oxygenated monoterpenes (9.5%). The group of hydrocarbons (63.2%) was predominant in the concrete, followed by diterpenes (22.9%) and oxygenated hydrocarbons (11.6%). The chemical profile of the resinoid was dominated by alkaloids (34.6%, nicotine being the single compound), followed by aliphatic hydrocarbons (18.7%) and oxygenated hydrocarbons (15.7%).

**Figure 2:**
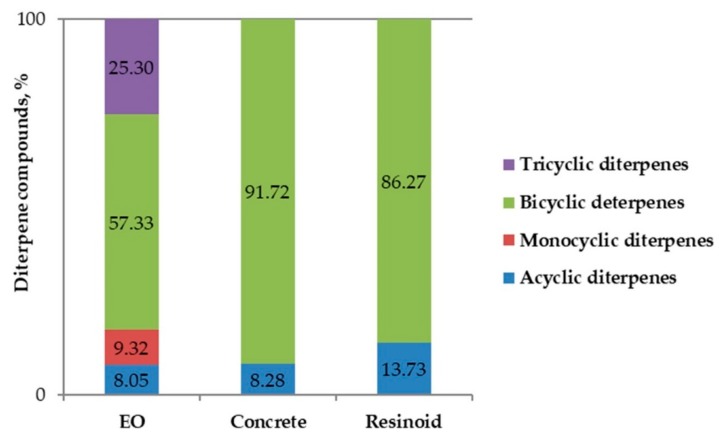
Diterpene profile of the EO and extracts of *N. glutinosa* leaves (total diterpene content = 100%).

As seen in Table 2, diterpenes (diterpenoids) were a major group of constituents not only in the EO (61.5% of the identified composition), but also in the concentrated extraction products. Diterpenes are a highly heterogenous group of compounds, all containing a C20 skeleton formed from the condensation of four isoprene units, which can be further divided into various sub-groups. The classification of the identified diterpene representatives in this study (Figure 2) was carried out depending on their skeletal core, into the five or six basic groups of diterpenes, i.e., linear (acyclic), monocyclic, bicyclic, tricyclic, tetracyclic, and pentacyclic or macrocyclic diterpenes. As seen in Figure 2, no representatives of the diterpenes with four or more rings were identified in either of the products. Concrete and resinoid contained only acyclic and bicyclic diterpenes, while the EO had a more diverse diterpene structure.

The bicyclic labdanoid sclareol, a diterpene alcohol, was of particular interest in this study, as it is a considered a characteristic metabolite of *N. glutinosa*. Indeed, sclareol was one of the major components of *N. glutinosa* EO, concrete and resinoid, found at concentrations of 3.6%, 14.2% and 6.9% of TIC, respectively (Table 1). As has already been stated, the presence of sclareol, together with the related labdane-type diterpenes in *N. glutinosa* supports the examination of the species as a one potentially important for the fragrance and cosmeceutical industries. Therefore, this study assessed the antimicrobial activity of the three aromatic products and pure sclareol against a set of medicinally and food important yeast and bacterial strains in an attempt to extend the knowledge about sclareol biological activities. The results, however, showed very limited activity of the products against the test-microorganisms (the respective inhibition zones (IZ), if any, were with diameters insignificantly larger than those of the discs in the agar diffusion test), so only the data from the antimicrobial tests of the pure substance sclareol are presented in Table 3. These results showed that only *Pseudomonas* bacteria, *P. putida,* and *P. aeruginosa* were completely resistant to sclareol, while the rest of the test-microorganisms were significantly susceptible (Table 3). Sclareol was most effective against the *Candida* spp. in the study. Meanwhile, in *C. albicans*, *C. glabrata*, *C. parapsilosis*, and *C. tropicalis*, the diameters of the IZs (14.2÷16.5 mm) were statistically equal to those of the control antibiotic Fluconazole (14.7÷16.6 mm). Respectively, those were the microbial strains affected by the minimal dilution concentrations in the study (MIC, MFC), although the concentration values were considerably higher than those of the positive control. With regard to bacteria, the highest intensity of sclareol activity was registered against *S. aureus*, with an IZ diameter of 14.2 mm and MIC/MBC of 512 µg/mL. The least susceptible were the Gram-negative Bacteria *S. abony* (IZ of 9.5 mm) and *E. coli* (IZ of 10.1 mm). The rest of the bacterial strains, *B. cereus, P. mirabilis,* and *P. vulgaris,* had similar susceptibility to sclareol, with IZs from 10.5 mm to 11.2 mm.

## 3. Discussion

It is known that EO bearing plants are traditionally used by the fragrance industry to obtain natural products concentrating the fragrance of the plant material. These products are all standardized, commercially recognized and produced on a relatively large base. They include EOs obtained almost exclusively by steam distillation and aromatic products obtained by extraction, i.e., absolutes, concretes, resinoids, aromatic waters, supercritical fluids extracts, pomades, and some others [33,34,35,36,37]. Since *N. glutinosa* has not yet been considered a source of natural aromatic products, in this study, we adapted the established processing technology [33,34] in order to obtain the EO, concrete, and resinoid of *N. glutinosa* leaves, and to identify the chemical profile of these products. It should be noted that only the volatile and semi-volatile composition (by GC-MS analysis) were investigated in this first report on the subject, as this is the most common approach for the characterization of plant-derived aromatic products, thus allowing for the comparative discussion and evaluation of results. The analysis of the non-volatile fractions of the aromatic products would certainly reveal additional aspects of their composition, which could be a potential focus of our future research on the species.

The results from the study suggested that in terms of aromatic product yields, *N. glutinosa* leaves were a plant material suitable for processing. The yield of the EO, 0.23 ± 0.50% DW, was in the range cited in previous reports on *N. tabacum* (0.2–1.5%) [31]. The yield of concrete, 1.56 ± 0.13% DW, was also comparable to the yield of concrete obtained from *N. tabacum* “Krumovgrad 90” variety of oriental tobacco (1.6%) [32]. In turn, the yield of resinoid, 10.6 ± 0.09% DW, was lower than that reported for various types and origins of common tobacco, e.g., 12%–17% [31] or 14%–29% [32], but still reasonably high.

There were no previous reports about obtaining and characterization of the EO, concrete and resinoid from *N. glutinosa* leaves, so it was hard to make direct parallels based on the data from the GC-MS analysis. Our results, however, supported previous findings stating that each of the aromatic products, common to the fragrance industry, i.e., EO, concrete, and resinoid, isolated from a given plant matrix, has a unique composition shaped by the factors involved in the isolation process, namely temperature, duration, selectivity of the solvent, and others [33]. The results also confirmed our previous observations about the differentiation of the aromatic products obtained from other *Nicotiana* species, different types and varieties of *N. tabacum* [32,38,39] and *N. alata* [40]. As seen in Table 1, only the EO of *N. glutinosa* contained carotenoid-related fragrance compounds, e.g., α-ionone (1.6%), 3,4-dehydro-β-ionone (2.1%), and trans-β-damascenone (1.6%), which was similar to our previous results on *N. tabacum* aromatic products [39] and the influence of pH, temperature, and duration of hydrodistillation on carotenoid degradation. The presence of phthalic acid esters in the EO (diisobutyl phthalate, dibutyl phthalate), substances with diverse toxicity profiles, was apparently a consequence of their presence in the plant material, as stated previously for other medicinal plants and EOs [41,42,43]. Moreover, evidence about phthalates identified in common tobacco (*N. tabacum*) and tobacco extracts was also documented in several publications [44,45,46,47]. Since the accumulation of phthalates and other plasticizer residues in the roots and the aerial parts of the plants occurs through different transfer routes [41,42,43], additional investigations are needed to identify the growing environment for *N. glutinosa* that would eliminate those contaminants from the final products. An interesting finding was the identification of the triterpene squalene in the concrete and resinoid, although not as a major component (1.6% and 2.9%, respectively). Squalene is a natural linear triterpene (with six isoprene units) found in shark liver oil (2300–8400 mg/100 g oil) and in the oils of several plants, such as amaranth (6000–8000 mg/100 g), olives (150–747 mg/100 g), soybean (1.2–180 mg/100 g), grape seeds (2.7–14.1 mg/100 g), etc., and it is associated with various nutritional and medicinal benefits [48]. Squalene, being a natural constituent of human skin, has a broad use in topically applied cosmetic and pharmaceutical products, due to its expressive emollient, hydrating, antioxidant, and other skin-protective properties [48,49].

The results about the chemical profile of the EO confirm previous findings that terpene compounds are decisive for the properties of the EOs, together with oxygenated aliphatics and phenyl propanoids [33,37]. Several major and minor fragrance compounds could have contributed to the olfactory profiles of the EO and extracts, such as *n*-hexanoic, 4-methylhexanoic, and octanoic acids (described with cheesy, fatty, sour, fruity odor), isophorone (cooling, woody, sweet, green, cedarwood, tobacco odor), neral and geranial (sweet, citrus, lemon odor), solanone and megastigmatrienones 1–4 (tobacco, fruity, winey odor), hexahydrofarnesyl acetone (herbal, jasmine, celery, woody odor), and farnesyl acetone (fruity, winey, creamy, floral odor) among others (Table 1). The results from the olfactory evaluation supports the eventual interest in *N. glutinosa* aromatic products, as contemporary fragrance industry is searching for distinct tobacco notes in masculine perfumery and cosmetics, for green notes in unisex products, and for balsamic and honey-like notes in fragrances for women, especially those of the “brunette” type. The relative distribution of chemical compounds supported the findings that polar solvents (ethanol) readily extracted plant alkaloids (34.6% share in *N. glutinosa* resinoid), while the distillation from an acidic medium (in the case of the EO) or the use of non-polar solvents (n-hexane, for concrete) resulted in minimal alkaloid transition to the final product [32,38,40]. The high alkaloid concentration in *N. glutinosa* resinoid, together with the presence of some compounds with antioxidant potential, suggested that future investigation of the aromatic product might prove its potential for use in hair-treatment cosmetic products (gels, lotions, etc.), in accordance with previous findings about the hair-growth stimulating effect of tobacco alkaloid-rich extracts, via the inhibition of 5-α-reductase activity [50]. As anticipated, the share of hydrocarbons (aliphatic and oxygenated) in the composition of the two extraction products was considerable (responsible for about 75% of the concrete and for about 35% of the resinoid), but not in that of the hydrodistilled EO [33,34,35,36,37]. Aliphatic hydrocarbons determined the appearance of the extraction concentrates (both were viscous masses, light or dark brown in color), but they are not carriers of the characteristic odor. All three aromatic products from *N. glutinosa* leaves, however, revealed a significant share of the fragrance-related terpene derivatives, namely—of oxygenated monoterpenes and diterpenes. The fragrance importance and the bioactivities associated with *Nicotiana* diterpenes (cembranoids, labdanoids) rationalized a more detailed insight into the diterpene profile [51,52] of the aromatic products of *N. glutinosa* leaves (Figure 2).

Acyclic diterpenes (constituting 8.1%, 8.3%, and 13.7% of the diterpene fraction of the EO, concrete, and resinoid, respectively) were represented by verticillol (C_20_H_34_O; in the EO) and phytol (C_20_H_40_O; in the concrete and resinoid). Phytol is probably the most abundant acyclic isoprenoid in nature. It is a major constituent of many EOs and plant extracts (including those of *N. tabacum*), and is widely used in perfumery and cosmetics (as emollient, masking and perfuming agent), with an odor described as floral, balsamic, powdery, waxy, and of low strength. Cembrene (thunbergen, C_20_H_32_) was the only monocyclic diterpene identified in the study, constituting 9.3% of the diterpenes in the EO; described with a wax-like odor of low intensity. In turn, tricyclic diterpenes were found only in the EO, and not in the extracted aromatic products. These included four pimarane diterpenes, with a 25.3% share in the total diterpene content. The group of bicyclic diterpenes had the largest share in the diterpene fraction (57.3% in the EO, 91.7% in the concrete and 86.3% in the resinoid) and was represented by the largest number of individual compounds. Manool (C_20_H_34_O), sclarene (C_20_H_32_) and manoyl oxide (C_20_H_34_O) were responsible for 23.8%, 14.1% and 13.6% of the total diterpene content in the EO, respectively. Manool finds limited use in perfumery as an individual isolate, however, being structurally related to the odorous components of ambergris and to sclareol, it is considered very important for the supply of ambergris substitutes for perfumery. The odor of manool is described as delicate, woody, dry-sweet, extremely tenacious with a typical ambergris-like undertone, and that of sclarene as a woody-type, powerful, green, terpenic, earthy, warm, amber, and clary sage. Sclareol was the only diterpene found in all three aromatic products of *N. glutinosa*, although its share in the EO (5.9%) was not as impressive as that in the concrete and resinoid (66.8% and 86.3% of the diterpene content, respectively). These results supported previous findings that the predominant diterpene alcohols in *N. glutinosa* were sclareol and manool [8,10,11]. Our results were consistent with the data about sclareol transfer to the EO and aromatic products of clary sage (*Salvia sclarea*) [53], considering that sclareol is almost non-distillable with steam, but its good solubility in alcohol, water, and other solvents facilitates its extraction. Sclareol possesses a delicate tobacco-amber odor, with sweet, balsamic, woody, weedy notes, and is a recognized starting material for the synthesis of Ambrox^®^ (Firmenich) and other sustainable substitutes of ambergris [54,55,56,57]. The waxy, musky-sweet-earthy-odored ambergris is highly appreciated and widely used as a fixative in fine perfumery, but its original supply depends on an endangered and protected animal species, sperm whales, and therefore investigations on alternative supply routes are relevant. Beside its perfume-fixating value, sclareol is also identified with a number of beneficial activities, such as antifungal, antimicrobial, growth-regulating, anti-inflammatory, and others [9,11,29,58]. Therefore, the results about the high content of sclareol in the two concentrated aromatic products of *N. glutinosa*, and in particular in the concrete, may be considered very promising in terms of potential future use and larger-scale production. As the obtaining of concrete is the first step of producing plant absolutes (the second stage includes cold extraction of concrete with ethanol to remove precipitated fractions) [33,34,35,36], it makes rational the obtaining and characterization of *N. glutinosa* absolute as well, which is set as an objective for our future work.

An important aspect in the discussion of the possible future use of *N. glutinosa* aromatic products in perfumery and cosmetics is the presence of chemical structures, which may cause skin sensitivity, irritations and other symptoms of allergy response. Moreover, the results from this study (Table 1) revealed that *N. glutinosa* leaf EO and extracts did not contain any of the 26 individual fragrance substances identified as allergens in humans, which must be indicated in the list of ingredients if exceeding 0.001% in leave-on products and 0.01% in rinse-off products [35,59]. Therefore, the results from this study might be a good starting point for future investigation on the aromatic products of *N. glutinosa*, similar to those performed on *N. tabacum*, in order to assess their safety as cosmetic products ingredients, in compliance with the provisions of Regulation (EC) No. 1223/2009 (The Cosmetics Regulation) [60], which defines the use of EO, natural plant extracts and their single compounds in cosmetic products. The results from this study are expected to validate *N. glutinosa* leaf EO and extracts as candidates to be included in the EC cosmetic ingredient database (CosIng).

In line with these considerations, we tested the antimicrobial activity of *N. glutinosa* aromatic products against a set of microorganisms, representing Gram-positive and Gram-negative bacteria and yeasts, responsible for different infections and skin problems. We also analyzed the activity of the individual component common to the three aromatic products, sclareol, a substance of importance to the fragrance industry. As stated earlier, sclareol was a characteristic component of *N. glutinosa* leaf derived aromatic products, and data about its antimicrobial and insecticidal activity have been documented previously [9,11,29,58]. Regrettably, the products showed very limited activity against the test-microorganisms in this study. These results were probably due to some synergistic/antagonistic component interactions within the products or to problematic solubility, as the phytocomplexes contained agents potent of antimicrobial activity (Table 1). Sclareol, however, demonstrated antimicrobial activity against the tested microorganisms, with the exception of the two *Pseudomonas* strains, which were completely resistant. The results in Table 3 showed that sclareol was most effective against the *Candida* yeasts in the study, namely *C. albicans*, *C. glabrata*, *C. parapsilosis*, and *C. tropicalis*, while the intensity of antimicrobial action (diameter of the IZs) was statistically equal to that of the control antibiotic (Fluconazole). As seen from Table 3, Gram-positive bacteria, *B. cereus* and *S. aureus*, were generally inhibited more effectively compared with the Gram-negative bacteria, *E. coli*, *S. abony*, *P. mirabilis*, and *P. vulgaris*. These observations were consistent with the principle difference in the intrinsic resistance of the two bacteria categories, defined by the different structure and permeability of cell walls. Given the increased *Candida* incidence, especially in immunocompromised people, as well as the issues with the growing resistance of *Candida* spp. to common treatments, these findings might be considered satisfactory. Thus, the results from this study expand knowledge on sclareol, as new data were provided on sclareol activity tests against some microorganisms that were not previously reported.

The results about the yield and the chemical composition of natural aromatic products derived from *N. glutinosa* leaves reveal its cultivation potential as an EO crop for obtaining fragrance materials, but also for obtaining sclareol and concentrated diterpene extracts rich in sclareol. The economics of clary sage (*S. sclarea*), sclareol, sclareolide, and Ambrox^®^ production worldwide [39,61] demonstrate a constant increasing trend, but production hardly matches the demand of the fragrance industry. In turn, *N. glutinosa* was reported to produce over 3200 kg per ha of fresh leaf biomass (at a planting density of 17,600 plants per ha, a standard planting density for flue-cured tobacco) [18]. These data, combined with the sufficiently high yield of aromatic products from *N. glutinosa* leaves demonstrated in this study, namely EO (0.23%), concrete (1. 6%) and resinoid (10.6%), provide justification for the commercial production and processing of this species.

## 4. Materials and Methods

### 4.1. Plant Material

**Figure 3:**
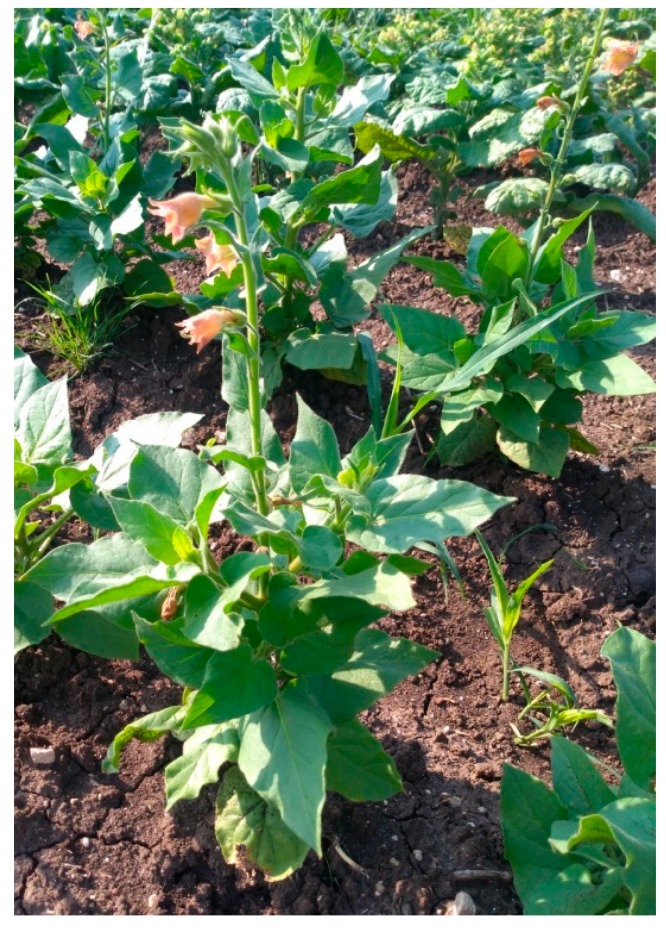
Plants of *N. glutinosa* at the experimental field (photo by authors).

*N. glutinosa* plants (Figure 3) were grown experimentally on the fields of Tobacco and Tobacco Products Institute (part of Bulgarian Agricultural Academy), situated in the region of Plovdiv, southern Bulgaria (42°04′55.2″ N 24°42′16.8″ E). The soil was hummus-carbonate (rendzina), with an organic matter content of 2.31%, total nitrogen content of 0.21%; mobile forms of phosphorus (Р_2_О_5_) of 14.85 mg/100 g soil, available potassium (K_2_O) of 67.5 mg/100 g soil, and a pH of 8.2. The vegetation period was June to September 2018. Due to the drought susceptibility of the species, additional irrigation was carried out twice during vegetation. All leaves were successively collected (picked by hand at maturity), and then sun cured in strings for about two weeks. Cured leaves were stored in an air-conditioned environment (in cardboard boxes) until processing.

In the sample preparation step, leaves were oven-dried (40 °C; 6 h), ground in a laboratory mill and sieved. The moisture content was determined by drying (103 ± 2 °C) to constant weight [62], and all results are presented on a dry weight (DW) basis.

### 4.2. Obtaining and Analysis of Aromatic Products

The essential oil (EO) was obtained by hydrodistillation (from a strongly acidified medium, pH 2) using a modified laboratory glass apparatus of the British Pharmacopoeia [63]. Freshly collected EO was dried over anhydrous sodium sulfate and stored in tightly closed dark vials at 4 °C until analysis.

Resinoid was obtained by extraction with 95% ethanol (FILLAB, Bulgaria) under the following conditions: twofold batch extraction for 2.5 h and 2 h; temperature 70 °C; raw material: solvent ratio of 1:10. The solvent was completely removed by evaporation on a rotary vacuum evaporator at a water bath temperature of 55 °C [62].

Concrete was obtained by extraction with *n*-hexane (Sigma-Aldrich, Steinheim, Germany) under the following conditions: twofold batch extraction for 1 h and 0.5 h; temperature 30 °C; raw material:solvent ratio of 1:10. The solvent was completely removed by evaporation using a rotary vacuum evaporator at a temperature of 35 °C [62].

### 4.3. Olfactory Evaluation of the EO and Extracts

Olfactory analysis of the aromatic products from *N. glutinosa* leaves (EO, concrete and resinoid) was performed by a panel consisting of three certified perfumers. The products were pre-conditioned to room temperature, dropped on an odor strip and evaluated individually by each of the panelists. The individual results were synchronized to obtain consistent odor description [33].

### 4.4. Chemical Composition of the EO and Extracts

#### 4.4.1. GC-MS Analysis of the Extracts (Concrete and Resinoid)

Each sample (50 µL) was derivatized with 100 µL pyridine (Sigma-Aldrich) and 100 µL *N,O*-bis(trimethylsilyl)trifluoroacetamide (BSTFA; Supelco), in an incubation for 45 min at 70 °C. Then 150 µL chloroform were added and the solution (1 µL) was injected into a system consisting of a 7890А gas chromatograph (Agilent Technologies Inc., Santa Clara, CA, USA) coupled with a 5975С mass selective detector (Agilent Technologies Inc.) and an HP-5 ms column (length 30 m, internal diameter 0.32 mm, film thickness 0.25 µm). The temperature program was set from 40 °C (held for 0 min) to 230 °C, at an increase step of 5 °C/min (held for 10 min at the maximal temperature); injector and detector temperatures were 250 °C; carrier gas was helium at a flow rate of 1.0 mL/min; mass detector scan range was *m*/*z* = 50–550; the split mode was at a 5:1 ratio.

#### 4.4.2. GC-MS Analysis of the EO

The samples (50 µL) were diluted with 350 µL *n*-hexane and analyzed in the same system and column as described above. The operational conditions were as follows: starting temperature 60 °C (held 0 min), increased to 300 °C at 5 °C/min (held at 300 °C for 10 min); injector and detector temperatures 250 °C; carrier gas helium at 1.0 mL/min flow rate; mass detector scan range *m*/*z* = 40–500; injected sample volume 1 µL in a flow split mode (split ratio 1:10).

GC-MS detected volatiles were identified by comparison with data from mass spectra libraries ([64]; NIST 08 database; own libraries). The retention (Kovat′s) indices were calculated using a standard *n*-alkane calibration mixture (C_8_–C_40_) in hexane. The quantity of the identified compounds was expressed as percentage of TIC, after normalization of the recorded peak areas.

### 4.5. Antimicrobial Activity of Sclareol

The in vitro antimycotic activity of sclareol (Sigma-Aldrich) against a set of medicinally important *Candida* strains was determined by a disc agar diffusion test, according to reference method M44-A2 [65]. The following test-cultures were used: *C. albicans* АТСС 10231, *C. glabrata* ATCC 90030, *C. parapsilosis* clinical isolate, and *C. tropicalis* NBIMCC 23. The positive control was Fluconazole (FLC, 25 μg), used under the same test conditions. The diameter of the inhibition zones around the discs was measured with an antibiotic zone scale PW 297 (HiMedia Laboratories Ltd., Mumbai, India), to an accuracy of 1.00 mm, and was considered proportionate to the intensity of the antimicrobial activity of the investigated compound.

The in vitro determination of the minimal inhibitory concentration (MIC) and the minimal fungicidal concentration (MFC) of sclareol against the yeasts of genus *Candida* was carried out by the serial broth dilution susceptibility test, according to reference method М27-А3 [66]. MIC was defined as the lowest dilution concentration at which no development of test-microorganisms in the discs was visually detected. MFC was defined as the lowest concentration at which no growth of single colonies was detected. Fluconazole (FLC MD072, 0.016÷256 μg/mL) was used as a positive control, and the MIC of the antimycotic was determined with HiComb™ MIC Test (HiMedia Lab. Ltd., India), according to producer’s instructions.

The in vitro antibacterial activity of sclareol was determined by the above-described procedure, according to Standard M2-A9 [67] for the disc agar diffusion test and Standard M7–A7 [68] for the serial broth dilution test, respectively. The bacterial test-cultures in the study were *Bacillus cereus* ATCC 11778, *Escherichia coli* ATCC 8739, *Salmonella abony* ATCC 6017, *Pseudomonas putida* ATCC 12633, *Pseudomonas aeruginosa* ATCC 9027, *Staphylococcus aureus* ATCC 6538, *Proteus mirabilis* ATCC 14153, and *Proteus vulgaris* ATCC 13315. The positive control used to test the susceptibility of the microorganisms were antibiotic discs of Ciprofloxacin (Cp, 10 μg/disc) (HiMedia Lab. Ltd., India).

All test-microorganisms were from the collection of the Department of Biochemistry and Microbiology, Plovdiv University “Paisii Hilendarski”, Bulgaria.

### 4.6. Statistics

All experiments were performed in a threefold repetition, and data were presented as mean value ± standard deviation. The statistical significance of differences was assessed by ANOVA and Tukey’s multiple comparison test (*p* < 0.05).

## 5. Conclusions

Natural aromatic products from *N. glutinosa* leaves of Bulgarian origin, essential oil (EO), concrete, and resinoid were obtained and analyzed. To the best of our knowledge, this is the first report on *N. glutinosa* EO, concrete, and resinoid characterization. The results from the GC-MS analysis of the aromatic products revealed their potential for use in the fragrance industry, as they all were rich in terpene and other aroma-active compounds. Further, it was demonstrated that the yields of the aromatic products from *N. glutinosa* were sufficiently high, comparable to those of other EO bearing plants. To the best of our knowledge, there is no evidence about any attempts for a larger-scale industrial production of *N. glutinosa*, or for its industrial processing, but the results from this study provide arguments in favor of the importance of this species, and open the door for future investigations.

## Figures and Tables

**Table 1:** Volatile composition (GC-MS) of essential oil (EO) and extracts from *N. glutinosa* L. leaves.

No	Compounds	RI ^1^	Content, % of TIC ^2^		
			EO ^3^	Concrete	Resinoid
1.	*n*-Hexanoic acid	975	0.13 ± 0.00 ^4^	nd ^5^	nd
2.	4-Methylhexanoic acid	1009	0.12 ± 0.00	nd	nd
3.	Isophorone	1118	0.17 ± 0.00	nd	nd
4.	Neral ((Z)-3,7-dimethyl-2,6-Octadienal)	1238	nd	nd	0.53 ± 0.01
5.	Octanoic acid tms ^6^	1258	nd	3.88 ± 0.02	nd
6.	Geranial ((E)-3,7-dimethyl-2,6-Octadienal)	1267	nd	nd	0.10 ± 0.00
7.	*n*-Nonanoic acid	1268	0.15 ± 0.00	nd	nd
8.	4-[(2E)-2-Butenyl]-1,2-dimethylbenzene	1312	0.43 ± 0.01	nd	nd
9.	Dehydro-ar-ionene (Naphthalene, 1,2-dihydro-1,1,6-trimethyl-)	1333	0.55 ± 0.01	nd	nd
10.	α-Ionone	1340	1.62 ± 0.01	nd	nd-
11.	Nonanoic acid tms	1355	nd	0.20 ± 0.01	nd
12.	Nicotine	1366	0.18 ± 0.00	0.46 ± 0.01	32.92 ± 0.16
13.	trans-β-Damascenone	1369	1.60 ± 0.02	nd	nd
14.	Solanone	1374	1.46 ± 0.02	nd	6.85 ± 0.03
15.	Naphthalene, 1,2-dihydro-1,5,8-trimethyl-	1377	0.62 ± 0.00	nd	nd
16.	3,4-Dehydro-β-ionone ((3E)-4-(2,6,6-Trimethyl-1,3-cyclohexadien-1-yl)-3-buten-2-one)	1457	2.11 ± 0.01	nd	nd
17.	Malic acid tms	1501	nd	0.22 ± 0.01	nd
18.	Lilial (3-(4-(tert-Butyl)phenyl)-2-methylpropanal)	1528	nd	nd	0.31 ± 0.01
19.	*n*-Hexyl benzoate	1545	1.21 ± 0.01	nd	nd
20.	Megastigmtrienone 1	1559	nd	0.84 ± 0.02	nd
21.	Megastigmtrienone 2	1582	nd	0.30 ± 0.02	nd
22.	Megastigmtrienone 3	1629	nd	1.12 ± 0.07	nd
23.	Megastigmtrienone 4	1656	nd	0.26 ± 0.01	nd
24.	Hexahydrofarnesyl acetone	1831	6.01 ± 0.03	0.30 ± 0.01	6.51 ± 0.03
25.	Tetradecanoic acid tms	1841	nd	0.41 ± 0.02	nd
26.	Diisobutyl phthalate	1856	17.61 ± 0.09	nd	nd
27.	Dihydroxyacetone dimer tms	1857	nd	0.52 ± 0.03	0.61 ± 0.01
28.	Sclareol oxide	1876	nd	3.67 ± 0.04	nd
29.	Isopimara-9(11),15-diene	1905	2.60 ± 0.02	nd	nd
30.	Dibutyl phthalate	1912	3.46 ± 0.02	nd	0.90 ± 0.01
31.	Farnesyl acetone	1912	nd	0.40 ± 0.03	nd
32.	Cembrene (Thunbergen)	1937	5.58 ± 0.04	nd	nd
33.	Sclarene	1973	8.42 ± 0.07	nd	nd
34.	Manoyl oxide	1994	8.11 ± 0.06	nd	nd
35.	Hexadecanoic acid tms	2039	nd	1.25 ± 0.02	nd
36.	Manool	2056	14.23 ± 0.08	0.50 ± 0.01	nd
37.	Verticillol	2090	4.82 ± 0.04	nd	nd
38.	Podocarp-7-en-3-one, 13β-methyl-13-vinyl-	2100	4.43 ± 0.03	nd	nd
39.	Pimara-7,15-dien-3-one	2104	3.43 ± 0.02	nd	nd
40.	Podocarp-7-en-3β-ol, 13β-methyl-13-vinyl-	2127	4.68 ± 0.03	nd	nd
41.	Phytol tms	2163	nd	1.76 ± 0.01	1.09 ± 0.01
42.	α-Linolenic acid	2168	nd	0.62 ± 0.01	nd
43.	Sclareol	2222	3.55 ± 0.03	14.20 ± 0.09	6.85 ± 0.04
44.	3-α-acetoxy-Manool	2236	nd	0.78 ± 0.06	nd
45.	3-α-hydroxy-Manool	2286	nd	0.35 ± 0.03	nd
46.	Octadecanoic acid tms	2340	nd	1.44 ± 0.02	nd
47.	*n*-Tetraicosane	2400	nd	1.85 ± 0.02	0.67 ± 0.01
48.	*n*-Pentacosane	2500	nd	1.34 ± 0.02	0.42 ± 0.01
49.	*n*-Hexacosane	2600	nd	1.50 ± 0.02	0.92 ± 0.01
50.	*n*-Heptacosane	2700	nd	1.27 ± 0.02	1.44 ± 0.01
51.	Diacylglycerol	2780	nd	nd	0.52 ± 0.01
52.	*n*-Octacosane	2800	nd	5.10 ± 0.04	0.41 ± 0.01
53.	Squalene	2812	nd	1.63 ± 0.02	2.92 ± 0.02
54.	*n*-Nonacosane	2900	nd	6.96 ± 0.05	0.91 ± 0.01
55.	*n*-Triacontane	3000	nd	10.65 ± 0.09	0.87 ± 0.01
56.	*n*-Hentriacontane	3100	nd	3.88 ± 0.02	3.07 ± 0.02
57.	α-Tocopherol	3136	nd	nd	8.20 ± 0.03
58.	*n*-Dotriacontane	3200	nd	5.48 ± 0.06	0.72 ± 0.01
59.	β-Stigmasterol	3226	nd	nd	2.08 ± 0.01
60.	*n*-Tritriacontane	3300	nd	6.17 ± 0.05	0.63 ± 0.01
61.	*n*-Tetratriacontane	3400	nd	4.09 ± 0.03	1.56 ± 0.01
62.	*n*-Pentatriacontane	3500	nd	3.15 ± 0.02	1.71 ± 0.01
63.	*n*-Hexatriacontane	3600	nd	6.52 ± 0.03	1.37 ± 0.01
64.	*n*-Heptatriacontane	3700	nd	0.94 ± 0.01	2.73 ± 0.02
65.	Tridecanoin	3744	nd	nd	6.90 ± 0.05
66.	*n*-Octatriacontane	3800	nd	1.48 ± 0.02	0.35 ± 0.00
*Sum of the identified*			*97.28*	*95.49*	*95.07*

^1^ RI—retention (Kovat’s) index; ^2^ identified at > 0.05% of TIC; ^3^ EO - essential oil; ^4^ data expressed as mean (*n* = 3) ± standard deviation; ^5^ nd—not detected or <0.05% of TIC; ^6^ tms—identified as trimethylsilyl derivatives; ^7^ the total sum of identified compounds = 100%.

**Table 2:** Distribution of the identified compounds by functional groups ^1^.

Compounds	Content, % of the identified		
	EO	Concrete	Resinoid
Hydrocarbons	nd	63.24	18.7
Oxygenated hydrocarbons	4.26	11.58	15.65
Oxygenated monoterpenes	9.49	0.31	7.51
Diterpenes	61.52	22.68	8.35
Triterpenes	nd	1.71	3.07
Phenyl propanoids	24.55	nd	9.9
Alkaloids	0.18	0.48	34.63
**Total**	**100**	**100**	**100**

^1^ The total sum of identified compounds = 100%.

**Table 3:** Antimicrobial activity of sclareol.

Test Microorganism	Sclareol			Positive Control ^4^	
	IZ ± SD ^1^	MIC ^2^	MBC/MFC ^3^	IZ ± SD	MIC
	mm	µg/mL	µg/mL	mm	µg/mL
*Bacillus cereus* ATCC 11778	11.2 ± 0.25	1024	1024	28.3 ± 0.30	0.125
*Escherichia coli* ATCC 8739	10.1 ± 0.11	1024	1024	21.0 ± 0.28	0.25
*Salmonella abony* ATCC 6017	9.5 ± 0.10	1024	1024	21.0 ± 0.28	0.25
*Pseudomonas putida* ATCC 12633	nd ^5^	nd	nd	10.3 ± 0.29	1.0
*Pseudomonas aeruginosa* ATCC 9027	nd	nd	nd	9.6 ± 0.17	1.0
*Staphylococcus aureus* ATCC 6538	14.2 ± 0.06	512	512	31.3 ± 0.29	0.125
*Proteus mirabilis* ATCC 14153	10.5 ± 0.11	1024	1024	19.6 ± 0.17	0.25
*Proteus vulgaris* ATCC 13315	11.1 ± 0.11	1024	1024	19.6 ± 0.17	0.25
*Candida albicans* АТСС 10231	16.5 ± 0.11	256	512	16.6 ± 0.29	0.25
*Candida glabrata* ATCC 90030	14.5 ± 0.30	256	512	15.0 ± 3.30	4.0
*Candida parapsilosis* clinical isolate	14.2 ± 0.10	256	512	15.7 ± 2.90	2.0
*Candida tropicalis* NBIMCC 23	15.2 ± 0.10	256	512	14.7 ± 3.90	4.0

^1^ IZ ± SD—diameter of inhibition zone ± standard deviation (*n* = 3); ^2^ MIC—minimal inhibition concentration; ^3^ MBC/MFC—minimal bactericidal/fungicidal concentration; ^4^ positive control—Ciprofloxacin (for bacteria) or Fluconazole (for *Candida* spp.); ^5^ nd—not detected.
